# Ba-induced phase segregation and band gap reduction in mixed-halide inorganic perovskite solar cells

**DOI:** 10.1038/s41467-019-12678-5

**Published:** 2019-10-15

**Authors:** Wanchun Xiang, Zaiwei Wang, Dominik J. Kubicki, Xueting Wang, Wolfgang Tress, Jingshan Luo, Jiahuan Zhang, Albert Hofstetter, Lijun Zhang, Lyndon Emsley, Michael Grätzel, Anders Hagfeldt

**Affiliations:** 1 State Key Laboratory of Silicate Materials for Architectures, Wuhan University of Technology, 430070 Wuhan, China; 2 Laboratory of Photomolecular Science, Institute of Chemical Sciences Engineering, Ecole Polytechnique Fedérale de Lausanne (EPFL), 1015 Lausanne, Switzerland; 3 Laboratory of Photonics and Interfaces, Institute of Chemical Sciences and Engineering, Ecole Polytechnique Fedérale de Lausanne (EPFL), CH-1015 Lausanne, Switzerland; 4 Laboratory of Magnetic Resonance, Institute of Chemical Sciences and Engineering, Ecole Polytechnique Fedérale de Lausanne (EPFL), CH-1015 Lausanne, Switzerland; 5 State Key Laboratory of Superhard Materials, Key Laboratory of Automobile Materials of MOE, and School of Materials Science and Engineering, Jilin University, Changchun, 130012 China; 6 Institute of Photoelectronic Thin Film Devices and Technology, Key Laboratory of Photoelectronic Thin Film Devices and Technology of Tianjin, Nankai University, 300350 Tianjin, China; 7 Present Address: Cavendish Laboratory, JJ Thomson Avenue, Kapitza Building K26, Cambridge, CB3 0HE UK

**Keywords:** Materials science, Materials for energy and catalysis, Solar cells

## Abstract

All-inorganic metal halide perovskites are showing promising development towards efficient long-term stable materials and solar cells. Element doping, especially on the lead site, has been proved to be a useful strategy to obtain the desired film quality and material phase for high efficient and stable inorganic perovskite solar cells. Here we demonstrate a function by adding barium in CsPbI_2_Br. We find that barium is not incorporated into the perovskite lattice but induces phase segregation, resulting in a change in the iodide/bromide ratio compared with the precursor stoichiometry and consequently a reduction in the band gap energy of the perovskite phase. The device with 20 mol% barium shows a high power conversion efficiency of 14.0% and a great suppression of non-radiative recombination within the inorganic perovskite, yielding a high open-circuit voltage of 1.33 V and an external quantum efficiency of electroluminescence of 10^−4^.

## Abstract (web summary)

Element doping has been proven a useful strategy to tune the properties of halide perovskites. Here Xiang et al. show that barium unexpectedly does not incorporate in perovskite lattice but induces phase segregation and bandgap reduction and inhibits non-radiative recombination.

## Introduction

Lead halide perovskites have the advantage of relatively high charge carrier diffusion lengths, high light harvesting efficiency, and band gap (*E*_g_) tunability, making them ideal materials for photovoltaic applications^1^. In 10 years, the certified power conversion efficiency (PCE) of perovskite solar cells (PSCs) has reached 25.2%, which is higher than that of polycrystalline silicon solar cells^2–6^. Despite these advances, the use of organic components (e.g., methylammonium and formamidinium) in perovskite materials generates stability concerns due to the volatility of the organic components^7–10^. Therefore, the development of all-inorganic perovskites for photovoltaic devices is currently extensively explored, as the replacement of organic components by their inorganic counterpart (Cs^+^) could solve the stability issue^11,12^.

Nevertheless, the inorganic lead halide PSCs are still facing large challenges in the improvement of PCE and phase stability, due to large *E*_g_ (1.7 eV to 2.3 eV) relative to the ideal *E*_g_ of ~1.3 eV for single-junction solar cells^13,14^, and instability of the cubic phase or black phase for I-based narrow band gap inorganic perovskites^15,16^. Element doping, especially on the lead site, has been proved to be a useful strategy to solve the above problems, as well as reduce the usage amount of toxic lead^17^. For example, Sn^2+^ and Ge^2+^, belonging to the same (IV) group as Pb^2+^, seem to be the most promising candidates to replace Pb^2+^, while also exhibiting band gap tunability^18,19^. However, the fast oxidation of Sn^2+^ and Ge^2+^ when exposed to ambient atmosphere limits the fabrication of Sn/Ge-based PSCs to inert environments^20^. Other divalent cations such as Mn^2+^, Sr^2+^, Eu^2+^, etc., have been explored as dopants for inorganic perovskites^15,21–24^, but they are generally at low doping ratios (typically around 5% on the lead site) and do not alter the band gap substantially. In addition, there is no consensus in terms of whether these cations are being incorporated into the perovskite lattice and the exact action mechanism of these dopants to improve the PSC performance is still under debate^25^.

Here we show that barium can be added in large quantities (the molar ratio of barium and lead up to 4:6 in the perovskite precursor solutions) during the synthesis of the inorganic CsPbI_2_Br perovskite, leading to the reduction of the perovskite band gap. The open-circuit voltage (*V*_OC_) of the inorganic PSCs with the addition of 20 mol% barium to the precursor solution is significantly improved to 1.33 V, yielding a PCE of 14.0%. To the best of our knowledge, this is the highest reported *V*_OC_ for all-inorganic PSCs with a band gap lower than 2 eV. Solid-state nuclear magnetic resonance (NMR) measurements evidence that there is no incorporation of barium into the perovskite lattice. Rather, barium segregates into barium-based non-perovskite phases with concomitant halide segregation. As a consequence, the perovskite phase is enriched in iodide, leading to changes in the lattice parameter and band gap compared with the undoped CsPbI_2_Br. The I-rich perovskite acts as the light-harvesting species and its band gap is linearly dependent on the amount of added barium, whereas the barium-based non-perovskite phases prohibit non-radiative recombination and are responsible for the high *V*_OC_.

## Results

### Perovskite characterizations and device performance

The perovskite precursor solutions were prepared by mixing CsI, PbI_2_, PbBr_2_, CsBr, and BaI_2_ in the required stoichiometric molar ratios in anhydrous dimethylsulfoxide (DMSO). The perovskite films were deposited from precursor solutions on mesoporous TiO_2_ substrates at a spinning speed of 3000 r.p.m. with the conventional one-step spin-coating method. The films were subsequently annealed at 280 °C for 10 min in dry air. For convenience, we refer to these films using their precursor solution stoichiometry: CsPb_1–*x*_Ba_*x*_I_2_Br (*x* = 0 to 1). Supplementary Fig. 1 shows the photographs of these perovskite films for different barium concentration. The color of the films gradually changes from dark into light brown as the barium fraction is increased. The pure CsBaI_2_Br film is colorless, confirming negligible absorption of visible light. Therefore, in the following we restrict ourselves to barium molar fraction *x* ≤ 0.4 for PSCs fabrication.

**Fig. 1:**
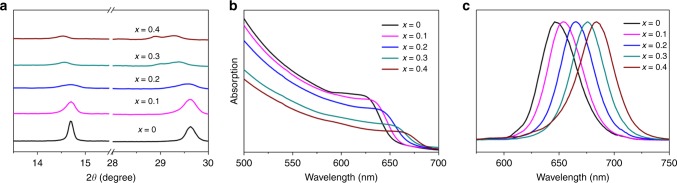
Materials characterization of CsPb_1−*x*_Ba_*x*_I_2_Br with *x* varies from 0 to 0.4 compositions. **a** XRD patterns, **b** UV-vis absorption, and **c** PL emission spectra

We first investigated the X-ray diffraction (XRD) of the CsPb_1–*x*_Ba_*x*_I_2_Br (*x* = 0 to 0.4) thin films. The XRD patterns are shown in Supplementary Fig. 2. The addition of barium resulted in a gradual decrease of the perovskite peak intensities (diagnostic peaks: 14.5^o^, 21.1^o^, 29.7^o^), which can still be reserved for *x* ≤ 0.4. A close scrutiny of the reflection peaks (Fig. 1a) revealed a noticeable shift to lower angles. According to the Bragg’s law, the low-angle shift reflects an expansion of the unit-cell volume^13^. Its effect on the optoelectronic properties was further examined by ultraviolet-visible (UV-vis) spectroscopy (Fig. 1b). We found a significant shift in the absorption onset to longer wavelength with increasing barium concentration. Meanwhile, a gradual decrease of the absorption intensity with constant film thickness indicates that the introduction of barium leads to a deterioration of the light-harvesting capacity. An analogous red-shift trend was observed in photoluminescence (PL) emission spectra (Fig. 1c). These results show that the introduction of barium alters the band gap of the parent CsPbI_2_Br perovskite.

**Fig. 2:**
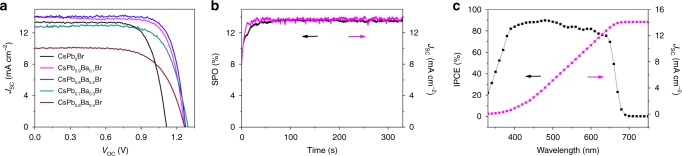
Photovoltaic performance of PSCs. **a** *J*–*V* performance of CsPb_1−*x*_Ba_*x*_I_2_Br (*x* = 0 to 0.4) based inorganic PSCs. **b** SPO and steady-state *J*_SC_ of the CsPb_0.8_Ba_0.2_I_2_Br-based PSCs. **c** IPCE and integrated *J*_SC_ of the CsPb_0.8_Ba_0.2_I_2_Br-based PSCs

The Ba-Pb mixed perovskites were then used to fabricate all-inorganic PSCs in the following device configuration: F-doped tin oxide (FTO) glass/compact-TiO_2_/mesoporous TiO_2_/CsPb_1−*x*_Ba_*x*_I_2_Br/2,2’,7,7’-tetrakis(*N*,*N*-di-p-methoxyphenyl-amine) 9,9’-spirobifluorene (Spiro-OMeTAD)/Au (for the cross-sectional scanning electron microscope (SEM) image of a complete device, please refer to Supplementary Fig. 3). The thickness of the perovskite layer is estimated to be around 170 nm. The photovoltaic parameters for *x* = 0, 0.1, 0.2, 0.3, and 0.4 are reported in Table 1 and the *J*–*V* curves are plotted in Fig. 2a. The champion PSC fabricated using CsPbI_2_Br (without barium) exhibits a *V*_OC_ of 1.12 V, a short circuit current (*J*_SC_) of 13.4 mA cm^−2^, a fill factor of 73.8%, yielding a PCE of 11.1%, which is comparable to those previously reported in the literature^20^. As the barium fraction increases to *x* ≥ 0.1, the *V*_OC_ enhanced dramatically from 1.12 V to around 1.27 V, which accounts for roughly 15% of the total improvement. The highest obtained *V*_OC_ from one of the best-performing devices fabricated using the CsPb_0.8_Ba_0.2_I_2_Br composition exhibited a value of 1.33 V (Supplementary Fig. 4). To the best of our knowledge, this is the highest reported *V*_OC_ for all-inorganic PSCs with a band gap lower than 2 eV. *J*_SC_ also shows a moderate increase as the barium concentration increases until *x* = 0.2, benefiting from the red-shifted absorption, in line with the UV-vis characterization. Further increase of the barium concentration to *x* *=* 0.3 leads to a dramatic decrease of *J*_SC_. The best PCE of 14.0% under one sun illumination was obtained for *x* = 0.2. The stabilized power output at maximum power point (MPP) tracking of the best device shows a value of 13.6% along with a steady-state current of 13.8 mA cm^−2^ (Fig. 2b). The incident photo-to-current conversion efficiency (IPCE; Fig. 2c) measurement demonstrates a broad absorption range from 640 to 380 nm with the absorption onset at 680 nm. The integrated *J*_SC_ of 14.1 mA cm^−2^ agrees also with the measured value.

**Table 1:** Photovoltaic parameters of CsPb_1−*x*_Ba_*x*_I_2_Br (*x* = 0 to 0.4) based inorganic PSCs

Barium molar fraction, *x*	*V*_OC_ (V)	*J*_SC_ (mA cm^−2^)	FF (%)	PCE (%)
0	1.12	13.4	73.8	11.1
0.1	1.27	13.9	75.3	13.3
0.2	1.28	14.0	78.2	14.0
0.3	1.29	12.8	70.8	11.7
0.4	1.27	10.1	66.1	8.4

These PSCs were illuminated under 100 mW cm^−2^ simulated sunlight.

**Fig. 3:**
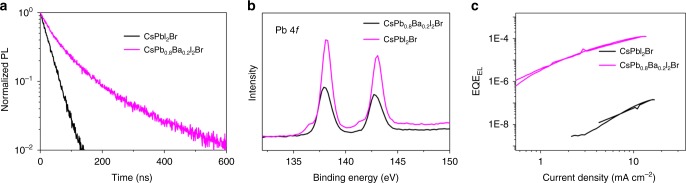
Non-radiative recombination analysis. **a** TRPL spectra of CsPbI_2_Br and CsPb_0.8_Ba_0.2_I_2_Br inorganic perovskite films. **b** Pb 4*f* spectra from XPS surface analysis of CsPbI_2_Br and CsPb_0.8_Ba_0.2_I_2_Br perovskite films. **c** EL measured during a *J*–*V* sweep from 0 to 1.6 V (1.4 V for CsPbI_2_Br-based PSCs) and back with a rate of 10 mV s^−1^

To elucidate the substantial enhancement of *V*_OC_ upon the addition of barium, we investigated the charge recombination behavior within the bulk perovskite by time-resolved PL (Fig. 3a). The plots show increased PL lifetimes upon barium addition. This result suggests suppression of non-radiative recombination pathways, consistent with the improved *V*_OC_ measured in barium-containing PSCs. In addition, the X-ray photoelectron spectra (XPS; Fig. 3b) of Pb 4*f* from CsPbI_2_Br show two small peaks at 136.9 eV and 141.7 eV, which are ascribed to the existence of metallic Pb. These two peaks completely disappear at 20% barium addition, implying that the introduction of barium can effectively suppress the formation of metallic Pb, which acts as non-radiative recombination centers and degrades PSCs performance. Thus, elimination of metallic Pb by barium can suppress the deep-level defects in PSCs^26^.

This spectacular improvement in *V*_OC_ was further elucidated using electroluminescence (EL) by studying the device with the champion *V*_OC_ shown in Supplementary Fig. 4. We can indeed observe an increase of the external EL quantum efficiency by three orders of magnitude when comparing the CsPb_0.8_Ba_0.2_I_2_Br with a device without barium (Fig. 3c). It reaches 10^−4^ for currents similar to *J*_SC_, which corresponds to a non-radiative loss of 240 mV^27^. Considering the theoretical maximum *V*_OC_ of 1.56 V for a solar cell based on a material with a band gap of 1.86 eV, we calculate a *V*_OC_ of 1.32 V, which is consistent with the experimental data^27^. Figure 3c shows that with the increase of the EL, the slope decreases, indicative of a reduced ideality factor, which approaches 2 for the barium-containing device^28^. These results indicate that the addition of barium into the CsPbI_2_Br precursor solution significantly reduces non-radiative recombination, which leads to a much improved *V*_OC_.

### Phase segregation analysis

**Fig. 4:**
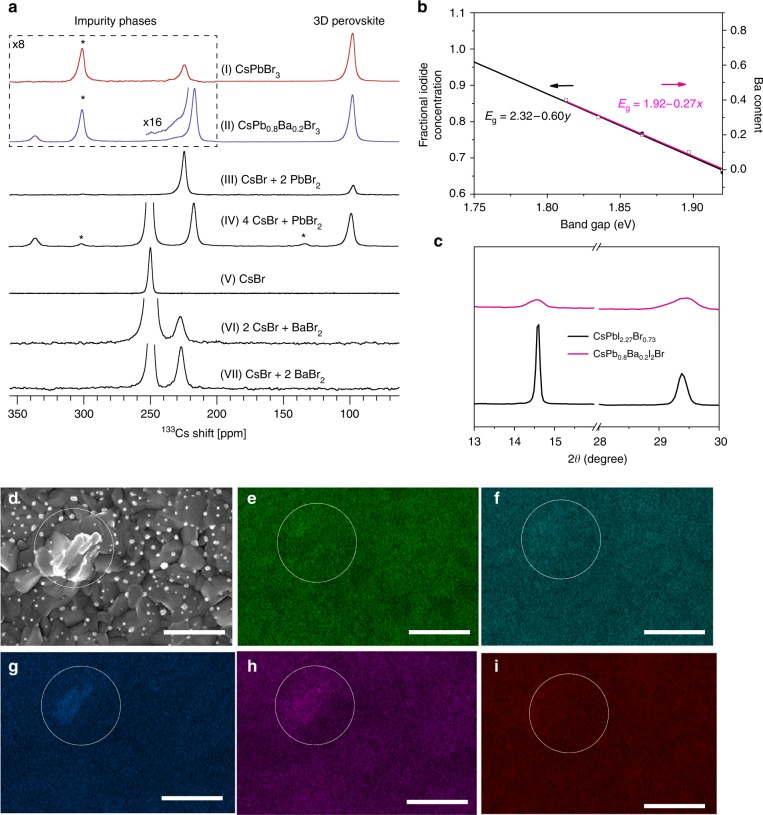
Phase segregation analysis. **a** ^133^Cs echo-detected solid-state MAS NMR spectra at 21.1 T, 298 K, and 24 kHz MAS of bulk mechanochemical compositions (I) CsPbBr_3_, (II) CsPb_0.8_Ba_0.2_Br_3_ (note that the formula designates the formal stoichiometry and does not correspond to a pure-phase material), (III) CsBr + PbBr_2_ (1:2), (IV) CsBr + PbBr_2_ (4:1), (V) neat CsBr, (VI) CsBr + BaBr_2_ (2:1), and (VII) CsBr + BaBr_2_ (1:2). Asterisks indicate spinning sidebands. The 200–350 p.p.m. range in **a** and **b** is magnified eight times to evidence the impurity phase. The inset in **b** shows a small amount of a barium-based non-perovskite impurity phase. **b** The fractional iodide concentration and barium concentration vs. the experimental band gaps. The linear fits are included in the figure. **c** XRD patterns of CsPbI_2.27_Br_0.73_ and CsPb_0.8_Ba_0.2_I_2_Br perovskite films. **d** an SEM image of a selected perovskite film area, the scale bar is 200 nm. Elemental distributions of CsPb_0.8_Ba_0.2_I_2_Br inorganic perovskite film by EDS elemental mapping are shown as: **e** distribution of Pb, **f** distribution of I, **g** distribution of Ba, **h** distribution of Br, and **i** distribution of Cs. The scale bars are 500 nm

We have recently shown that solid-state magic angle spinning (MAS) NMR is a suitable probe of the atomic-level microstructure in multi-component lead halide perovskites^29–31^. In particular, ^133^Cs can be used to probe cesium-containing phases in organic–inorganic and all-inorganic halide perovskites^30,32^. To evidence whether or not barium is incorporated substitutionally into the material described here, we carried out ^133^Cs MAS NMR measurements on barium-containing CsPbBr_3_ as a model compound (Fig. 4a). We chose the single-halide three-dimensional (3D) perovskite, as simultaneous introduction of bromides and iodides causes halide disorder leading to very broad ^133^Cs resonances (Supplementary Fig. 5)^32^. The reference mechanochemical CsPbBr_3_ material shows a nearly pure perovskite phase (100.5 p.p.m.; Fig. 4a (I)). It contains 4% of a secondary impurity phase (226.6 p.p.m.). The material with 20 mol% barium contains a perovskite phase (85%), whose spectrum is indistinguishable from that of CsPbBr_3_ (100.5 ppm) and impurity phases at 216.8 p.p.m. (14%) and 336.5 p.p.m. (1%). As the ^133^Cs shift of the main perovskite phase is, to within error, unchanged, it strongly suggests that there is negligible barium substitution of lead in the CsPbBr_3_. This is expected from the substantially larger ionic radius of Ba^2+^ (135 pm) compared with Pb^2+^ (119 pm). For further confirmation we have carried out fully relativistic NMR shift calculations confirming that lead substitution by barium would lead to a ^133^Cs shift with respect to CsPbBr_3_ of *ca* + 3 p.p.m. for each closest barium cation on an A- or B-site (Supplementary Table 1), which would lead to a clear change in the spectra. There are a number of known non-perovskite cesium lead bromides and cesium barium bromides with different stoichiometries. We have mechanochemically prepared 1:2 and 4:1 CsBr:PbBr_2_, and 2:1 and 1:2 CsBr:BaBr_2_ materials (Fig. 4a (III), (IV), (VI), (VII)). The 1:2 ratio of CsBr:PbBr_2_ yielded a mixture of CsPb_2_Br_5_ (224.6 ppm, 83%) and CsPbBr_3_ (100.5 p.p.m., 17%). The 4:1 ratio of CsBr:PbBr_2_ yielded a mixture of unreacted CsBr (57%, 249.6 p.p.m.), CsPbBr_3_ (17%, 100.5 p.p.m.), and two other phases, which we did not attempt to identify (216.8 p.p.m., 19% and 335.9 p.p.m., 6%). The two latter non-perovskite correspond perfectly to the impurity phases identified in the CsPb_0.8_Ba_0.2_Br_3_ composition (Fig. 4a (II)). Both CsBr:BaBr_2_ mixtures yielded a small amount of a new cesium barium bromide (227.0 p.p.m., 28% and 8% for the 1:2 and 2:1 ratios, respectively) and a corresponding amount of unreacted CsBr (Fig. 4a (V–VII)). The barium-based non-perovskite phase is present in the CsPb_0.8_Ba_0.2_Br_3_ composition (Fig. 4a (II), inset), albeit at a very low concentration (around 1%). Taken together, the ^133^Cs spectra show that there is no incorporation of barium into the model CsPbBr_3_.

Barium incorporation could also conceivably be probed using barium-137 NMR. Substitutional doping of barium on the A- or B-site should lead to a very symmetric (cubic or nearly cubic) ligand environment of barium and as a consequence to small electric-field gradient (EFG) tensors of the ^137^Ba nucleus and thus relatively narrow ^137^Ba NMR signals, as is the case for BaTiO_3_^33^. We have carried out fully relativistic EFG calculations for ^137^Ba incorporated on an A- and B-site of CsPbBr_3_ and confirmed that it should lead to a full width at half maximum (fwhm) of about 15 p.p.m. (1.5 kHz). We were unable to detect any ^137^Ba signal on the CsPb_0.8_Ba_0.2_Br_3_ composition over 12 h using an RF strength of 125 kHz, which should be sufficient to detect such a narrow resonance (Supplementary Fig. 6), suggesting that the barium species present in the composition have very large EFGs, consistent with asymmetric non-perovskite barium environments^34^. Taken together, the ^133^Cs and ^137^Ba NMR results are compelling evidence for no barium incorporation into the CsPbBr_3_ perovskite lattice. We expect them to carry over to the CsPbI_2_Br composition, as the chemical propensity of a cation to participate in substitutional doping has been shown to be halide-independent^29,35^. Thus, we conclude that the changes in band gap observed using optical spectroscopies are entirely due to the changes in the I/Br ratio in the final perovskite composition rather than barium incorporation.

To quantify the effect of barium addition on the band gap, we plot the band gaps as a function of barium concentration *x* in CsPb_1−*x*_Ba_*x*_I_2_Br. The band gaps are estimated from the wavelength of the maxima in the PL spectra in Fig. 1c. We observe a linear dependence between the band gaps and barium concentration *x* (Fig. 4b), which is described in Eq. (1):

1 $$E_{\mathrm{g}} = 1.92 - 0.27x$$

As the photoactive phase in the barium-containing material is still a cesium lead iodide-bromide, we prepared a series of CsPb(I_*y*_Br_1−*y*_)_3_ (*y* = 0–1) films with different Br/I ratio, recorded their PL peak wavelengths, and converted them to the corresponding band gaps. We plot these band gaps against the iodide fraction *y* and again observe a linear relationship as shown in Eq. (2):

2 $$E_{\mathrm{g}} = 2.32 - 0.60y$$

The empirical equation relating the barium fraction (*x*) in CsPb_1−*x*_Ba_*x*_I_2_Br and the iodide fraction (*y*) in CsPb(I_*y*_Br_1−*y*_)_3_ therefore has Eq. (3):

3 $$y = 0.45\,x + 0.667$$

From this equation, we find that the band gap of the composition formally corresponding to the formula CsPb_0.8_Ba_0.2_I_2_Br is identical to that of the CsPbI_2.27_Br_0.73_ perovskite. The XRD characteristic peaks of CsPbI_2.27_Br_0.73_ and CsPb_0.8_Ba_0.2_I_2_Br perovskites also both occur at 14.5° and 29.7° (Fig. 4c). To further clarify the advantage of barium addition on the photovoltaic performance of cesium lead halide PSCs, we prepared devices based on CsPbI_2.27_Br_0.73_ and 0.8CsPbI_2_Br-0.2CsI (i.e., CsPbI_2_Br doped with 20 mol% CsI) as references. Their efficiencies were 8.82% and 8.64%, respectively. Both types of PSCs show significantly lower *J*_SC_ and *V*_OC_ compared with CsPb_0.8_Ba_0.2_I_2_Br-based solar cells (Supplementary Fig. 7). Furthermore, the recorded *J*–*V* performance of the devices based on CsPbI_2_Br + *x*BaI_2_ (*x* = 0, 0.1, 0.2) shows that the addition of BaI_2_ increases the *V*_OC_ and the PCE (Supplementary Table 2 and Supplementary Fig. 8), indicating that Ba plays an important role in the improvement of photoelectric property of the corresponding devices. Besides, we found that no PL shift was observed for CsPbI_3_ or CsPbBr_3_ perovskites by barium addition (Supplementary Fig. 9a, b). Meanwhile, based on first-principles electronic structure calculations (Supplementary Figs. 10 and 11), we found that the band gaps of barium-doped perovskites CsPb_1−*x*_Ba_*x*_I_2_Br should increase, rather than decrease. This is expected from the much larger band gap of CsBaI_2_Br than that of CsPbI_2_Br and the type-I bands alignment between them. In addition, simulation of hypothetical alloys of CsPb_1−*x*_Ba_*x*_I_2_Br (*x* = 0, 0.1, 0.2, 0.3, 0.4, 1) gives positive formation energies with respect to decomposition into CsPbI_2_Br and CsBaI_2_Br (see Methods section and Supplementary Fig. 12). This indicates solid-solution miscibility gap and phase-separation behavior at low and room temperatures, consistent with the inability of Ba doped into perovskite lattice demonstrated experimentally. According to the Hume–Rothery rules^36^, the formation of solid solution needs similar atomic radii, same crystal structures, same electronegativities, and same valence states of constituted components. Except for the crystal structure and valence state, the substantial mismatches in the other two factors between Pb and Ba (ionic radii: 1.19 and 1.35 Å for Pb^2+^ and Ba^2+^, respectively; electronegativities) are responsible for the phase-separation behavior. Taken together, the results show that the shift of the XRD peak to higher angles in Fig. 1a and the red shifts of PL and UV-Vis spectra in Fig. 1b, c are entirely due to the change of the I/Br ratio in the perovskite, resulting from barium-induced anion exchange.

In order to map the distribution of the segregated phases in the perovskite films, we conducted Energy-dispersive spectroscopy (EDS)-based elemental mapping (Fig. 4e–i and Supplementary Fig. 13). The CsPb_0.8_Ba_0.2_I_2_Br perovskite film characterized by SEM (Fig. 4d) shows a uniform coverage of the perovskite on the substrate and an average crystal size of about 200 nm. The surface is covered with a large number of bright spots (in the white circle). EDS mapping shows that these bright parts contain higher ratio of Ba and Br than the rest, whereas the dark areas possess more I and Pb, which is verified by the EDS quantification (Supplementary Table 3**)**. These results are consistent with the phenomenon of phase segregation evidenced by solid-state NMR. Thus, the formal CsPb_1−*x*_Ba_*x*_I_2_Br stoichiometry does not represent a pure-phase material but a mixture of perovskite and barium-rich non-perovskite phases. The depth profile XPS analysis shows that Ba is located not only on the surface but also inside the whole CsPb_0.8_Ba_0.2_I_2_Br film (Supplementary Fig. 14). As Ba is well distributed across the perovskite layer and cannot be incorporated into the perovskite crystal lattice, its location is most likely at the surface and grain boundaries^37^.

### Perovskite film morphology and device stability

We also analyze the top-view film morphology by SEM after introducing barium during fabrication of CsPbI_2_Br on a mesoporous TiO_2_ substrate (Supplementary Fig. 15). A pinhole-free and dense pristine CsPbI_2_Br film was obtained by carefully controlling the deposition conditions, as described in the experimental section. Upon addition of 10% barium, the perovskite grains become well resolved and the grain boundaries become clear. However, bare TiO_2_ surface is visible underneath, evidencing incomplete coverage of the substrate. For a barium molar fraction *x* of 0.2, we observe full coverage of the substrate with closely packed grains and numerous dots on the film surface. Further increase in the barium content generates pinholes and makes the grain profile blurry, leading to less dense films. Apparently, morphology reconstruction takes place when barium is added into the composition, with *x* = 0.2 leading to optimal morphology for obtaining high performance solar cells.

To evaluate the stability of the complete devices, one of the best performing and unencapsulated CsPb_0.8_Ba_0.2_I_2_Br-based inorganic PSCs was illuminated under continuous 100 mW cm^−2^ white light-emitting diode (LED) irradiation in an N_2_ atmosphere with MPP tracking. The control CsPbI_2_Br-based PSC was monitored under identical conditions (Supplementary Fig. 16). The CsPb_0.8_Ba_0.2_I_2_Br-based device showed a slow decrease of PCE over time, maintaining ~80% of the initial efficiency after 450 h. During the same testing period, the PCE of CsPbI_2_Br-based PSC dropped dramatically to 40% of the initial value within 90 h and eventually < 20% after 350 h of illumination. These results indicate that the CsPb_0.8_Ba_0.2_I_2_Br-based PSCs demonstrate better stability than CsPbI_2_Br-based devices. As iodide-rich all-inorganic perovskites are known to be rather sensitive to moisture, we conclude that the enhanced stability of CsPb_0.8_Ba_0.2_I_2_Br-based PSCs is probably due to protection of the film surface with the barium-rich phases or defects reduction within the device^38^.

## Discussion

In conclusion, we report a function of adding barium to the all-inorganic CsPbI_2_Br perovskite and its use as an absorber layer in inorganic PSCs that achieves a PCE of 14.0% under one sun illumination. By applying solid-state NMR, we have shown that barium does not incorporate into the perovskite lattice. Rather, it segregates into barium-based non-perovskite phases. This in turn leads to a change in the iodide-to-bromide ratio in the photoactive perovskite layer. This atomic-level insight provides a full explanation of the changes observed in the optical spectra and XRD patterns. EL measurements indicate that the non-radiative recombination is effectively inhibited upon the addition of barium, yielding a much higher *V*_OC_ of 1.33 V and a high EL EQE value. The addition of barium also improves the quality of perovskite films and leads to an increase in PCE. Our study highlights that phase segregation is a critical design criterion, which should be taken into account and investigated for other additives.

## Methods

### Preparation of perovskite solutions

CsI (259.8 mg), 183.5 mg PbBr_2_, and 230.5 mg PbI_2_ were mixed with 1 ml anhydrous DMSO with continuous heating at 80 °C for 6 h until a completely clear CsPbI_2_Br solution was formed. BaI_2_ (391.2 mg) and 212.8 mg CsBr were dissolved into a mixture of 1.4 ml DMSO and formiamide (v:v = 1:1) to form a clear CsBaI_2_Br solution. The CsPb_1−*x*_Ba_*x*_I_2_Br (*x* = 0–1) perovskite solutions were prepared by mixing the above two solutions with appropriate ratio in a nitrogen-filled glovebox and alloyed overnight. The 1 M solutions of CsPbI_2.27_Br_0.73_, 0.8CsPbI_2_Br-0.2CsI, and CsPbI_2_Br + *x*BaI_2_ (*x* = 0.1, 0.2) were also prepared stoichiometrically.

### Device fabrication

The perovskite device was fabricated with the following procedure. The FTO conducting glass (Nippon Corp., Japan) with the square resistance of 10 Ω sq^−1^ was cleaned with 2% Hellmanex solution, acetone, and ethanol by sonication for 15, 10, and 10 min, separately. The 40 nm-thick TiO_2_ compact layer was deposited on top of cleaned FTO glass by spray pyrolysis at 450 °C from a precursor solution containing 0.6 ml titanium diisopropoxide bis(acetylacetonate) and 0.4 ml acetoacetate in 9 ml anhydrous ethanol. The 150 nm-thick mesoporous TiO_2_ layer was then deposited by spin-coating a diluted TiO_2_ paste (30 nm particle size, Dyesol) in absolute ethanol at the speed of 5000 r.p.m. for 10 s. These TiO_2_ films were dried at 80 °C for 5 min and further sintering process at 450 °C for 1 h under continuous dry air flow. After cooling down to ~100 °C, these films were immediately transferred into glovebox filled with dry air. The CsPb_1−*x*_Ba_*x*_I_2_Br (*x* *=* 0 to 1) perovskite films were prepared by spin-coating the aforementioned precursor solutions in a two steps program at 1000 r.p.m. and 3000 r.p.m. for 10 s and 30 s, respectively. The films were then left for 5 min before being annealed at 280 °C for 10 min. After cooling down to room temperature, the spiro-OMeTAD (Merck) chlorobenzene solution (90 mg ml^−1^) with 20.6 μl bis(trifluromethylsulfonyl)imide lithium salt (LiTFSI, Sigma-Aldrich, 520 mg ml^−1^ in acetonitrile) and 35.5 μl 4-tert-butylpyridine (t-BP, Sigma-Aldrich) was spun on top of the perovskite film at 4000 r.p.m. for 20 s. An 80 nm-thick Au back-contact metal electrode was finally thermal-evaporated to complete the device construction.

### X-ray diffraction

The perovskite films on mesoporous TiO_2_ substrates were prepared exactly as described in the device fabrication section. XRD patterns were acquired with a PANalytical Empyrean diffractometer in the transmission-reflection mode, using the Cu Kα radiation and the Ni β-filter.

### Scanning electron microscope

The perovskite films were deposited on mesoporous TiO_2_ substrates to exactly reflect the true film morphology. A high-resolution SEM (Zeiss Merlin) with in-lens detector was used for morphology characterization. EDS was carried out in SEM mode.

### Optical characterization

UV-vis measurements were performed on a Varian Cary 5. PL spectra were obtained with Fluorolog 322 (Horiba Jobin Yvon, Ltd) with the range of wavelength from 500 to 800 nm by exciting at 450 nm with a standard 450-W Xenon CW lamp. The samples were mounted at 60° and the emission recorded at 90° from the incident beam path. Time-resolved PL was performed using Fluorolog 322 spectrofluorometer (Horiba Jobin Yvon, Ltd). A NanoLED-405L (Horiba) laser diode (405 nm) was used for excitation. The samples were mounted at 60° and the emission collected at 90° from the incident beam path. The detection monochromator was set to 650 nm and the PL was recorded using a picosecond photodetection module (TBX-04, Horiba Scientific).

### Perovskite mechanosynthesis

Starting materials were stored inside a glovebox under argon. Perovskite powders were synthesized by grinding the reactants in an electric ball mill (Retsch Ball Mill MM-200 using a grinding jar (10 ml) and a ball (⌀10 mm) for 30 min at 25 Hz. The resulting powders were annealed at 280 °C for 10 min to reproduce the thin-film synthetic procedure. The amounts of reagents taken into the synthesis were as follows:

CsPb_0.8_Ba_0.2_Br_3_: 0.213 g CsBr (1.00 mmol), 0.059 g BaBr_2_ (0.20 mmol), and 0.294 g PbBr_2_ (0.80 mmol).

CsPbBr_3_: 0.213 g CsBr (1.00 mmol) and 0.367 g PbBr_2_ (1.00 mmol).

CsBr + 2 PbBr_2_: 0.107 g CsBr (0.50 mmol) and 0.367 g PbBr_2_ (1.00 mmol).

4 CsBr + PbBr_2_: 0.213 g CsBr (1.00 mmol) and 0.092 g PbBr_2_ (0.25 mmol).

2 CsBr + BaBr_2_: 0.213 g CsBr (1.00 mmol) and 0.149 g BaBr_2_ (0.5 mmol).

1 CsBr + 2 BaBr_2_: 0.107 g CsBr (0.50 mmol) and 0.297 g BaBr_2_ (1 mmol).

### Solid-state NMR measurements

Room temperature ^133^Cs (181.1 MHz) and ^137^Ba (100.0 MHz) NMR spectra were recorded on a Bruker Avance Neo 21.1T spectrometer equipped with a 3.2 mm CPMAS probe. ^133^Cs shifts were referenced to 1 M aqueous solution of CsCl, using solid CsI (*δ* = 271.05 p.p.m.) as a secondary reference^39^. ^137^Ba shift were referenced to solid BaZrO_3_ using BaO as a secondary reference (481 p.p.m.)^33^. Quantitative ^133^Cs spectra were acquired using a recycle delay of 450 s.

### Electronic structure calculations of Ba-doped CsPbI_2_Br perovskites

Electronic band structure calculations of Ba-doped CsPbI_2_Br perovskites were within the framework of density functional theory (DFT) by using plane-wave pseudopotential methods as implemented in the Vienna Ab initio Simulation Package^40,41^. The electron–ion interactions were described by the projected augmented wave pseudopotentials with the 6*s* (Cs), 5*s*, and 5*p* (Br), 5*s* and 5*p* (I), and 6*s* and 6*p* (Pb) electrons treated as valence electrons. We used the generalized gradient approximation of Perdew–Burke–Ernzerhof (PBE) form^42^ as exchange-correlation functional. The kinetic energy cutoff for the plane-wave basis of 300 eV was used and the *k*-point meshes of spacing were set to 2*π* × 0.04 Å^−1^ for electronic Brillouin zone integration. The structures of Ba-doped perovskites CsPb_1−*x*_Ba_*x*_I_2_Br (*x* = 0.03375, 0.0625, 0.125) were constructed through the 2 × 2 × 2, 2 × 1 × 2, 1 × 1 × 2 supercells of the CsPbI_2_Br unit cell, in which Pb atoms are randomly substituted by Ba atoms according to Ba concentration *x*. The structures are optimized through total energy minimization with the residual forces on the atoms converged to below 0.05 eV/Å. The spin–orbit coupling was included in the electronic structure calculations. Although the DFT-PBE approach is known to underestimate the band gaps of semiconductors due to the self-interaction error issue, but in the current case it is reliable to predict the correct trend on the calculated band gaps of Ba-doped perovskites CsPb_1−*x*_Ba_*x*_I_2_Br changing with Ba concentration *x*.

The hypothetical alloys of CsPb_1−*x*_Ba_*x*_I_2_Br (*x* = 0, 0.1, 0.2, 0.3, 0.4, 1) were simulated through the special quasirandom structure^43^ to mimic random disorder within a 200 atom supercell. The alloy formation energy is defined as Δ*H* = *E*(CsPb_1−*x*_Ba_*x*_I_2_Br) − *xE*(CsPbI_2_Br) − (1 − *x*)*E*(CsBaI_2_Br), where *E*(CsPb_1−*x*_Ba_*x*_I_2_Br), *E*(CsPbI_2_Br), and *E*(CsBaI_2_Br) refer to the free energy of CsPb_1−*x*_Ba_*x*_I_2_Br, CsPbI_2_Br, and CsBaI_2_Br phases, respectively. We assumed the 3D perovskite as the structure of CsBaI_2_Br to evaluate Δ*H*. The resulted value is positive, indicating a phase-separation condition. If there was the ground-state structure with the even lower energy of CsBaI_2_Br, we can expect that the Δ*H* would be even larger in the positive magnitude. Therefore, with the assumption of CsBaI_2_Br being 3D perovskite, our calculations give a reasonable evaluation of the phase-separation condition. The positive Δ*H* for all the Ba fractions indicates that there is no stable configuration for the alloyed perovskites at 0 K and phase-separation will occur. This may be attributed to the substantial ionic radius and electronegativity mismatch between Pb and Ba.

### X-ray photoelectron spectroscopy

The perovskite films were deposited on plain glass substrates as described above. XPS measurements were carried out using a PHI VersaProbe II scanning XPS microprobe (Physical Instruments AG, Germany). Analysis was performed using a monochromatic Al Kα X-ray source of 24.8 W power with a beam size of 100 µm. The spherical capacitor analyzer was set at 45° take-off angle with respect to the sample surface. The pass energy was 46.95 eV yielding a fwhm of 0.91 eV for the Ag 3*d* 5/2 peak. Curve fitting was performed using the PHI Multipak software.

### Photovoltaic performance

The PSCs were tested with a 450 W xenon light source (Oriel). The light intensity was calibrated by a silicon photodiode equipped with an IR-cutoff filter (KG3, Schott) and was recorded for each measurement. The current–voltage curves (*J*–*V* curves) of the devices were recorded by applying an external voltage bias with Keithley 2400 to give the corresponding current response. A black metal mask with an area of 0.16 cm^−2^ was applied to define the active area of the cells and to avoid the overestimation of the light input.

IPCE measurements were carried from the monochromatic visible photons, from Gemini-180 double monochromator Jobin Yvon, Ltd (UK), powered by a 300 W xenon light source (ILC Technology, USA) superimposed on a 1 mW cm^−2^ LED light. The monochromatic incident light was passed through a chopper running at 8 Hz frequency and the on/off ratio was measured by an operational amplifier.

### Stability

Stability measurements were performed with a Biologic MPG2 potentiostat under a full AM 1.5 Sun-equivalent white LED lamp. The devices were masked to 0.16 cm^2^ and were connected to a separate channel of a potentiostat. A continuous flow of dry nitrogen gas was flushed into the custom-built airtight weathering chamber as the sample holder. The devices were measured in situ with a MPP tracking routine under continuous illumination. The MPP was updated every 10 s by a standard perturb-and-observe method. A Peltier element in direct contact with the films was used to control the temperature of the devices. A Pt100 thermometer inserted between the Peltier element and the film was used to measure the temperature of the devices. Every 30 min a *J*–*V* curve was recorded, to track the evolution of individual *J*–*V* parameters. The setup is centrally controlled through a LabView interface allowing for automatic programming of experiments (temperature, illumination, atmosphere, and electronic measurements).

### Reporting summary

Further information on research design is available in the Nature Research Reporting Summary linked to this article.

## Supplementary information

Supplementary Information

Solar Cells Reporting Summary

Source Data

## Data Availability

The data that support the plots within this paper and other findings of this study are available in separate Supplementary Source Data Files in Supplementary Information section. All other relevant data are available from the corresponding authors upon reasonable request.
