# Supplementary material for: Ba-induced phase segregation and band gap reduction in mixed-halide inorganic perovskite solar cells
Source: Nat Commun. 2019 Oct 15;10:4686. doi: 10.1038/s41467-019-12678-5 (PMC6794321; doi:10.1038/s41467-019-12678-5)
Supplement: Supplementary file 1 — Supplementary Information [file 41467_2019_12678_MOESM1_ESM.pdf]

Supplementary Information

Ba-induced phase segregation and band gap reduction in mixed-halide inorganic perovskite solar cells

Xiang et al.

Supplementary Information

Ba-induced phase segregation and band gap reduction in mixed-halide inorganic perovskite solar cells

Wanchun Xiang[1,2,*]†, Zaiwei Wang[2]†, Dominik J. Kubicki[3,4, £], Xueting Wang[5], Wolfgang Tress[2], Jingshan Luo[3,5], Jiahuan Zhang[2], Albert Hofstetter[4], Lijun Zhang[5], Lyndon Emsley[4], Michael Grätzel[3], Anders Hagfeldt[2,*]

[1]State Key Laboratory of Silicate Materials for Architectures, Wuhan University of Technology, 430070, Wuhan, China.

[2]Laboratory of Photomolecular Science, Institute of Chemical Sciences Engineering, Ecole Polytechnique Fedérale de Lausanne (EPFL), 1015 Lausanne, Switzerland.

[3]Laboratory of Photonics and Interfaces, Institute of Chemical Sciences and Engineering, Ecole Polytechnique Fedérale de Lausanne (EPFL), CH-1015 Lausanne, Switzerland.

[4]Laboratory of Magnetic Resonance, Institute of Chemical Sciences and Engineering, Ecole Polytechnique Fedérale de Lausanne (EPFL), CH-1015 Lausanne, Switzerland.

[5]State Key Laboratory of Superhard Materials, Key Laboratory of Automobile Materials of MOE, and School of Materials Science and Engineering, Jilin University, Changchun 130012, China

[6]Institute of Photoelectronic Thin Film Devices and Technology, Key Laboratory of Photoelectronic Thin Film Devices and Technology of Tianjin, Nankai University, 300350, Tianjin, China.

†These authors contribute equally to this work.

*Correspondence: xiangwanchun@whut.edu.cn,

*Correspondence: anders.hagfeldt@epfl.ch.

£Current address: Cavendish Laboratory, JJ Thomson Ave, Kapitza Building K26, Cambridge CB3 0HE, United Kingdom.

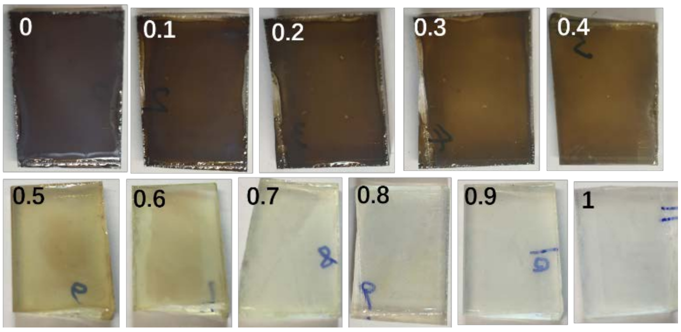

Supplementary Figure 1**.** Digital images of the $CsPb_{1-x}Ba_xI_2Br$ (x = 0 to 1) inorganic perovskite films on mesoporous TiO_2 substrates.

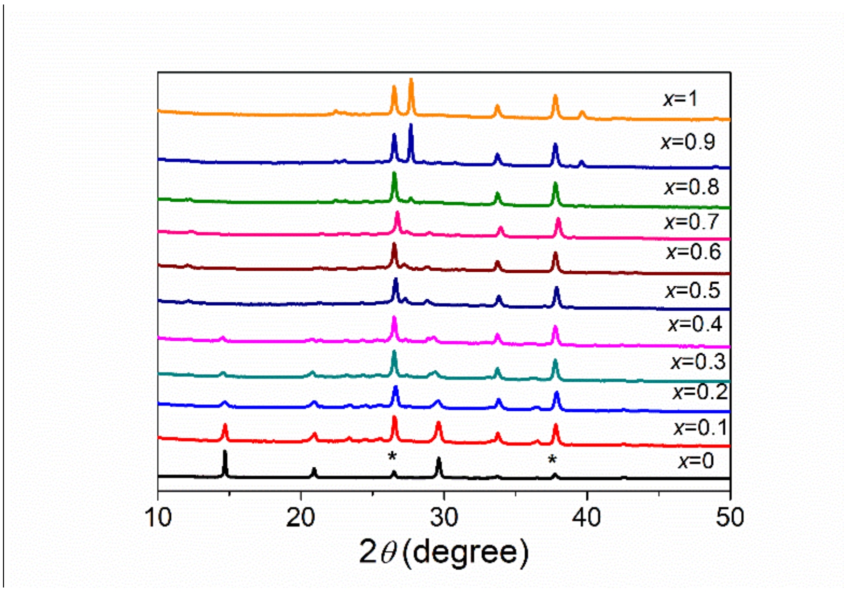

Supplementary Figure 2**.** The XRD patterns of $CsPb_{1-x}Ba_xI_2Br$ (x = 0 to 1) perovskites with different barium concentrations, * represents the FTO peaks.

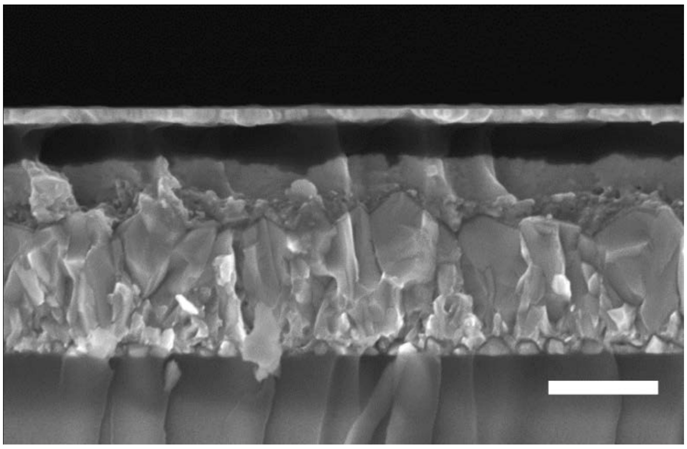

Supplementary Figure 3**.** Cross-sectional SEM image of a complete inorganic PSC. The scale bar is 500 nm.

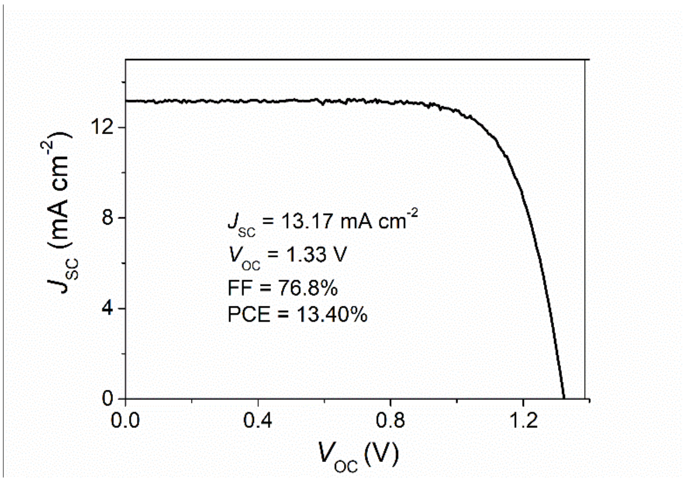

Supplementary Figure 4**.** *J*-*V* performance of $CsPb_{0.8}Ba_{0.2}I_2Br$-based inorganic PSCs with high V_{OC} measured under 100 mW cm^{-2} irradiation.

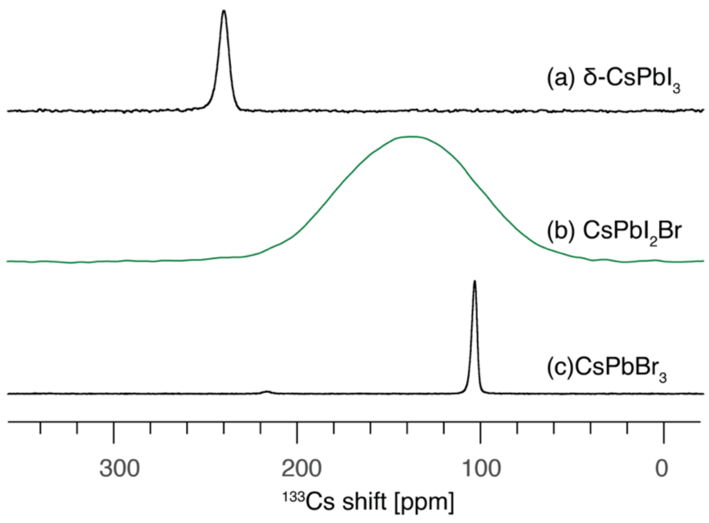

Supplementary Figure 5. ^{133}Cs echo-detected solid-state MAS NMR spectra at 11.7 T, 298 K and 20 kHz MAS of bulk mechanochemical compositions: a) δ-$CsPbI_3$, b) $CsPbI_2Br_3$, c) $CsPbBr_3$.

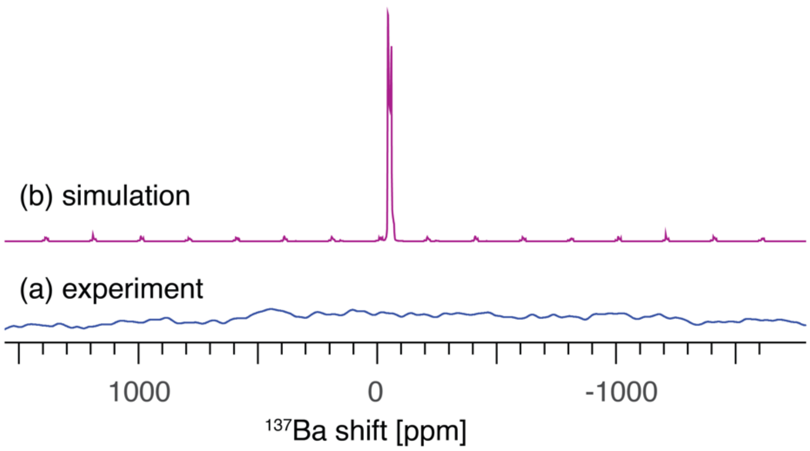

Supplementary Figure 6. a) ^{137}Ba echo-detected (CT-selective) solid-state MAS NMR spectrum at 21.7 T, 298 K and 20 kHz MAS of the bulk mechanochemical $CsPb_{0.8}Ba_{0.2}Br_3$ compositions. Similarly, no signal was detected with a non-selective echo. b) A simulated NMR spectrum using EFG parameters calculated by fully-relativistic DFT for Ba incorporated on a B-site of $CsPbBr_3$.

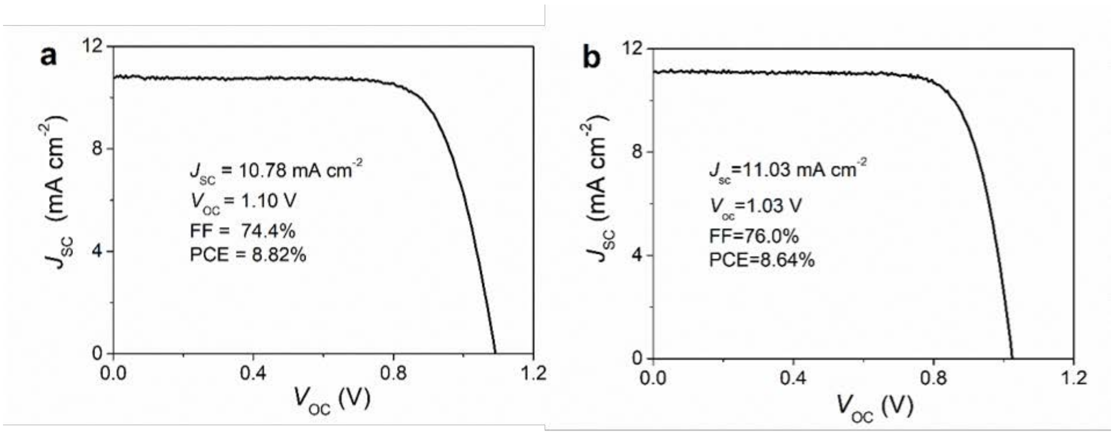

Supplementary Figure 7**.** *J*-*V* performance of a) $CsPbI_{2.27}Br_{0.73}$ and b) $0.8CsPbI_2Br$-$0.2CsI$ based inorganic PSCs under 100 mW cm^{-2} irradiation.

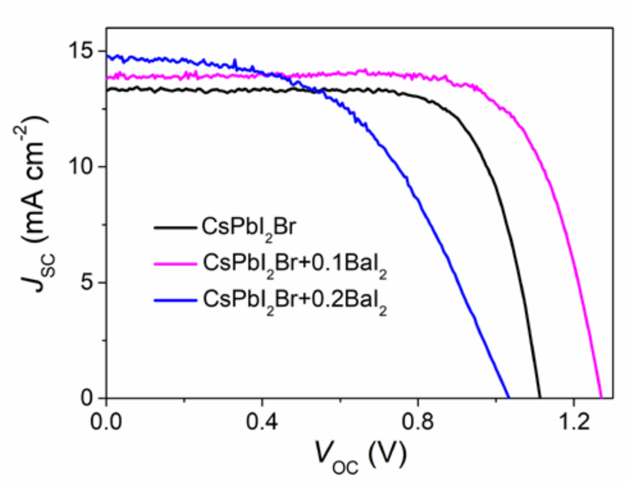

Supplementary Figure 8**.** *J-V* curves of $CsPbI_2Br$ based PSCs with different amount of BaI_2 as additive (Measurement was conducted under 100 mW cm^{-2} illumination with active area of 0.16 cm^{-2}).

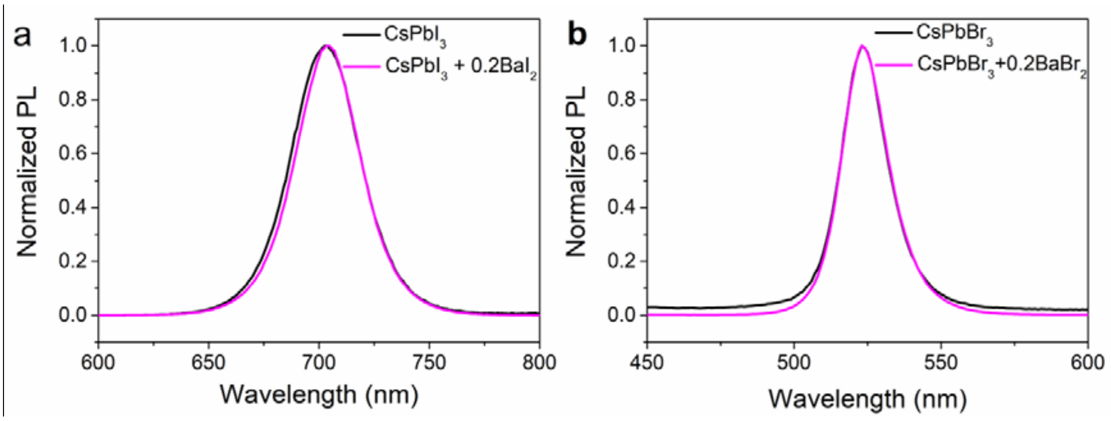

Supplementary Figure 9**.** PL spectra of a) $CsPbI_3$ and b) $CsPbBr_3$ inorganic perovskite films with and without barium.

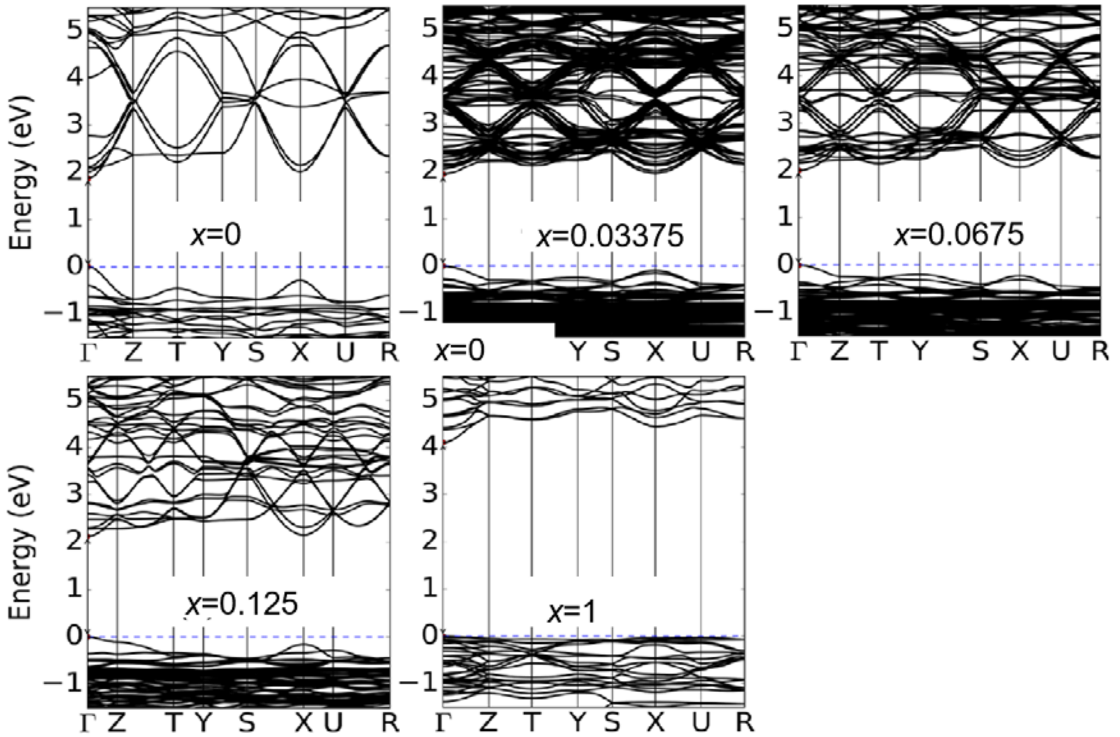

Supplementary Figure 10**.** Calculated electronic band structures of Ba-doped $CsPbI_2Br$ perovskites $CsPb_{1\text{-}x}Ba_xI_2Br$ with x = 0.03375, 0.0625, 0.125 with first-principles density functional theory approach assumed that barium was doped into the perovskite lattice. The lowest energy structure of $CsPb_{1\text{-}x}Ba_xI_2Br$ was used for the calculations.

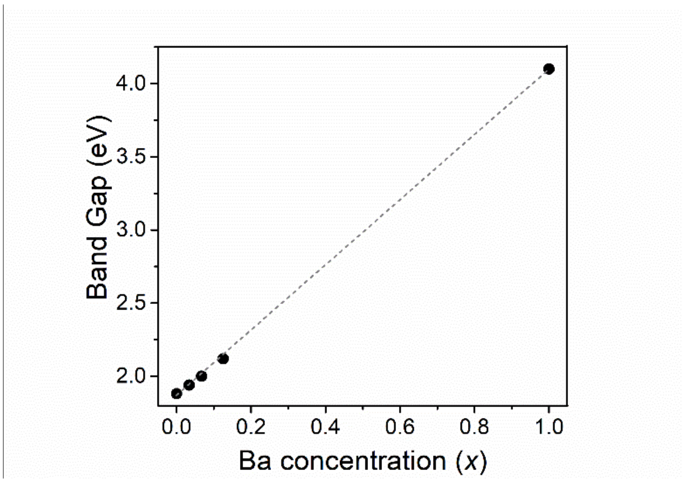

Supplementary Figure 11**.** Dependence of the calculated band gaps of the Ba-doped $CsPbI_2Br$ perovskites $CsPb_{1-x}Ba_xI_2Br$ on the barium concentration (x). The dot line represents linear fitting of the data.

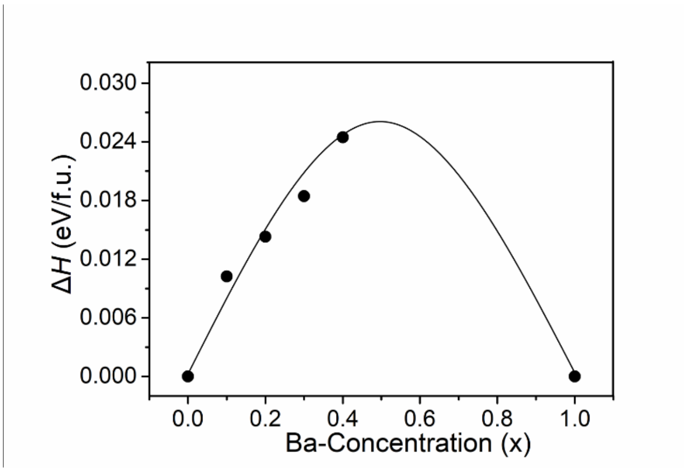

Supplementary Figure 12. Calculated formation energies of the alloyed perovskite $CsPb_{1-x}Ba_xI_2Br$ (x = 0, 0.1, 0.2, 0.3, 0.4, 1) by using the special quasirandom structure to mimic random disorder within a 200 atom supercell.

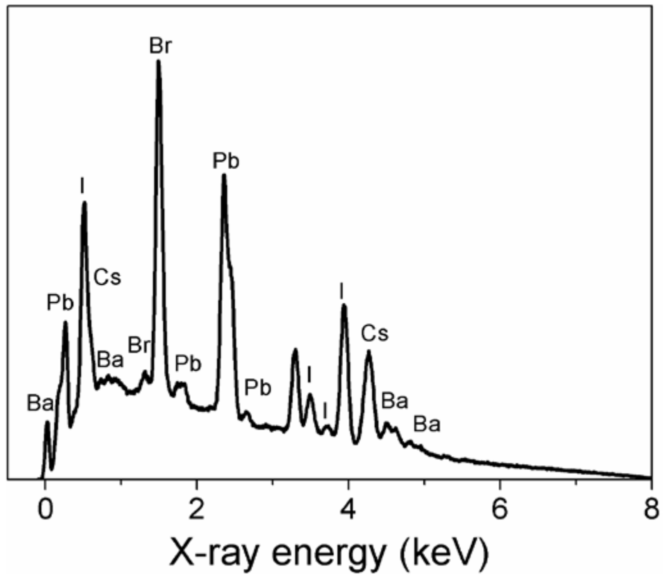

Supplementary Figure 13**.** Energy-dispersive spectroscopy (EDS) (EDS in SEM) elemental mapping of the $CsPb_{0.8}Ba_{0.2}I_2Br$ inorganic perovskite.

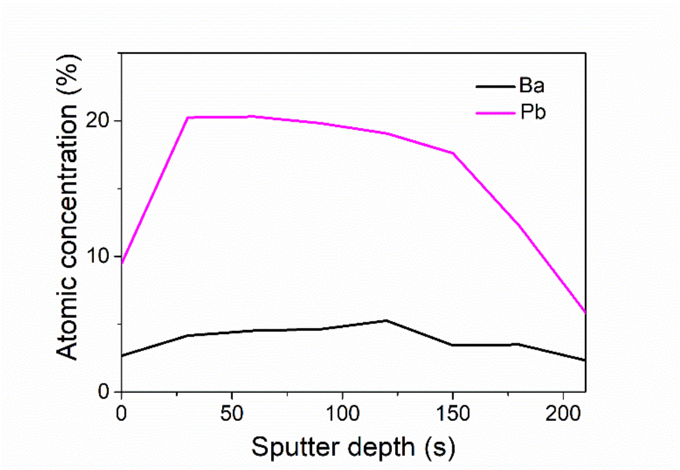

Supplementary Figure 14**.** Depth profile XPS of Pb and Ba in a $CsPb_{0.8}Ba_{0.2}I_2Br$ perovskite film.

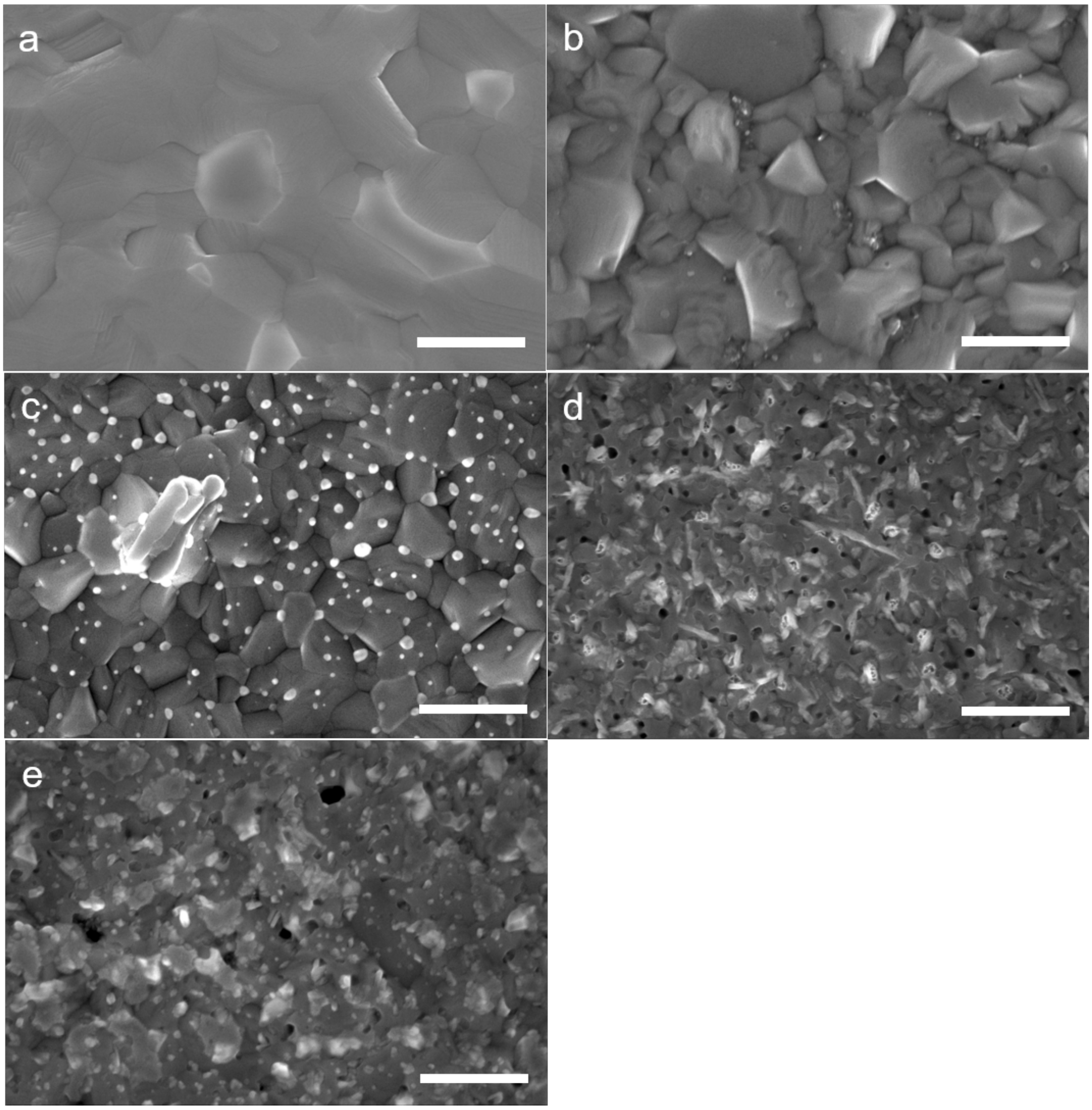

Supplementary Figure 15**.** SEM images of $CsPb_{1-x}Ba_xI_2Br$ with different incorporation amount of Ba (from a to e: x = 0, 0.1, 0.2, 0.3, 0.4, 0.5). The scale bars are 500 nm.

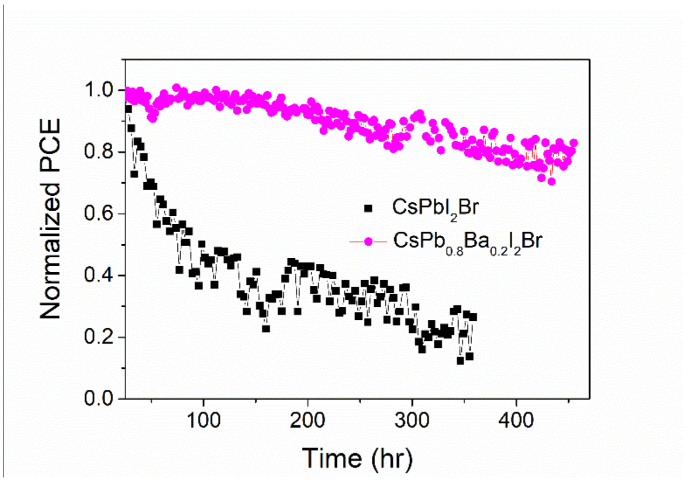

Supplementary Figure 16**.** Stability of the unencapsulated $CsPbI_2Br$ and $CsPb_{0.8}Ba_{0.2}I_2Br$ based PSCs under 100 mW cm^{-2} continuous irradiation.

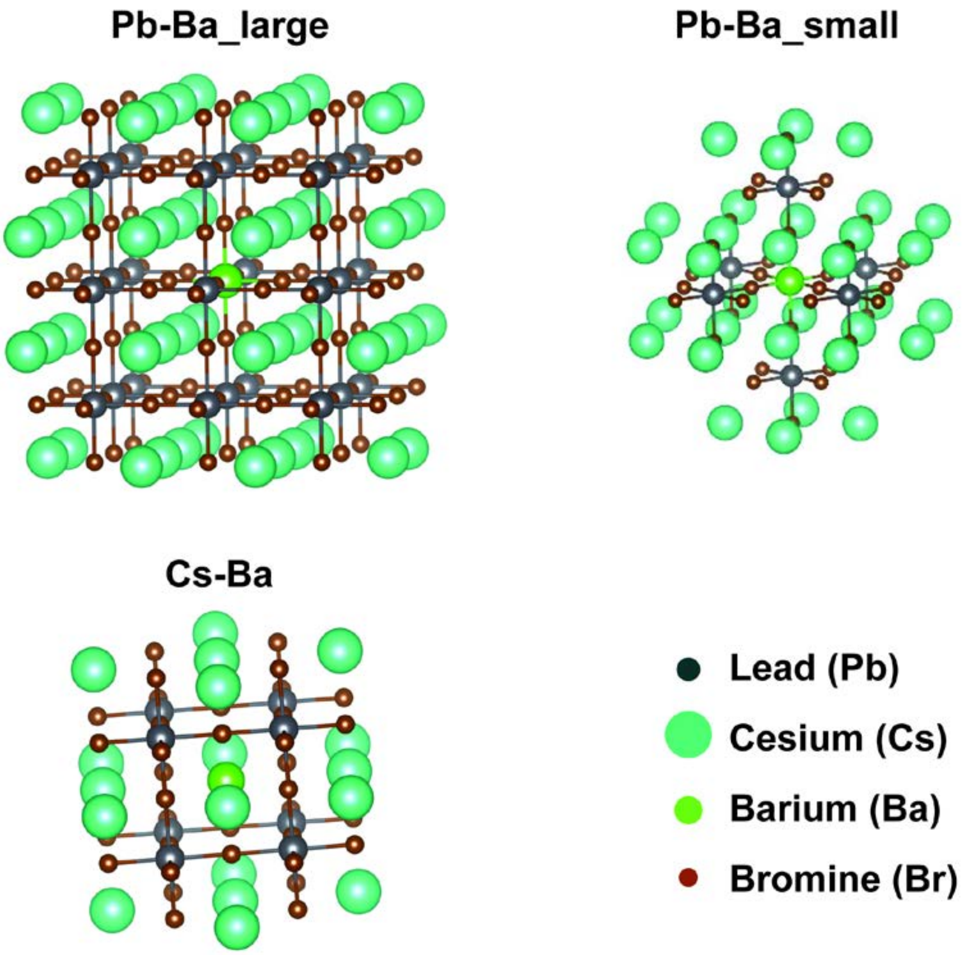

Supplementary Figure 17. $CsBaPbBr_3$ clusters used for DFT ^{137}Ba EFG tensor calculations

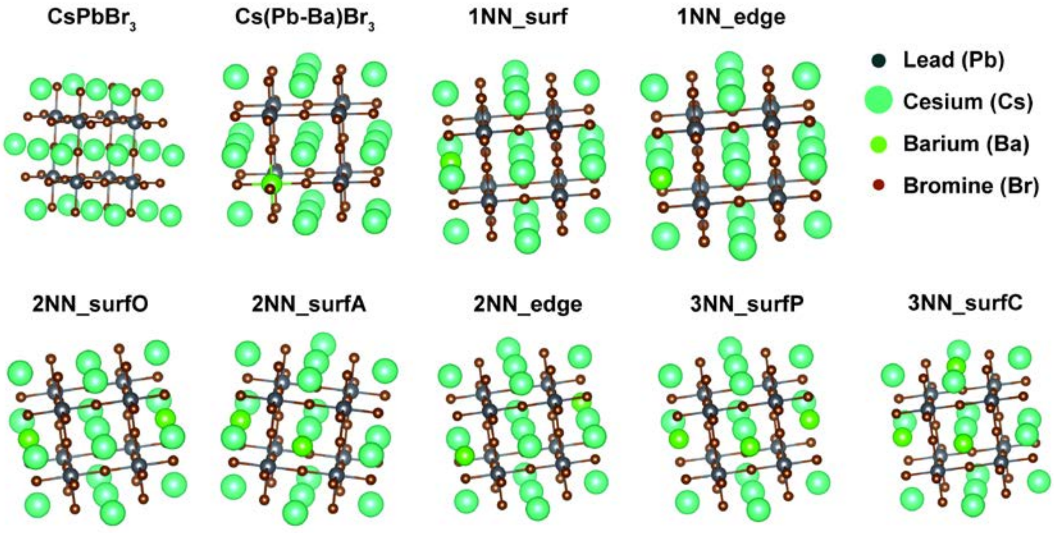

Supplementary Figure 18. Cubic (*Pm-3m*) $CsBaPbBr_3$ clusters used for DFT ^{133}Cs chemical shift calculations.

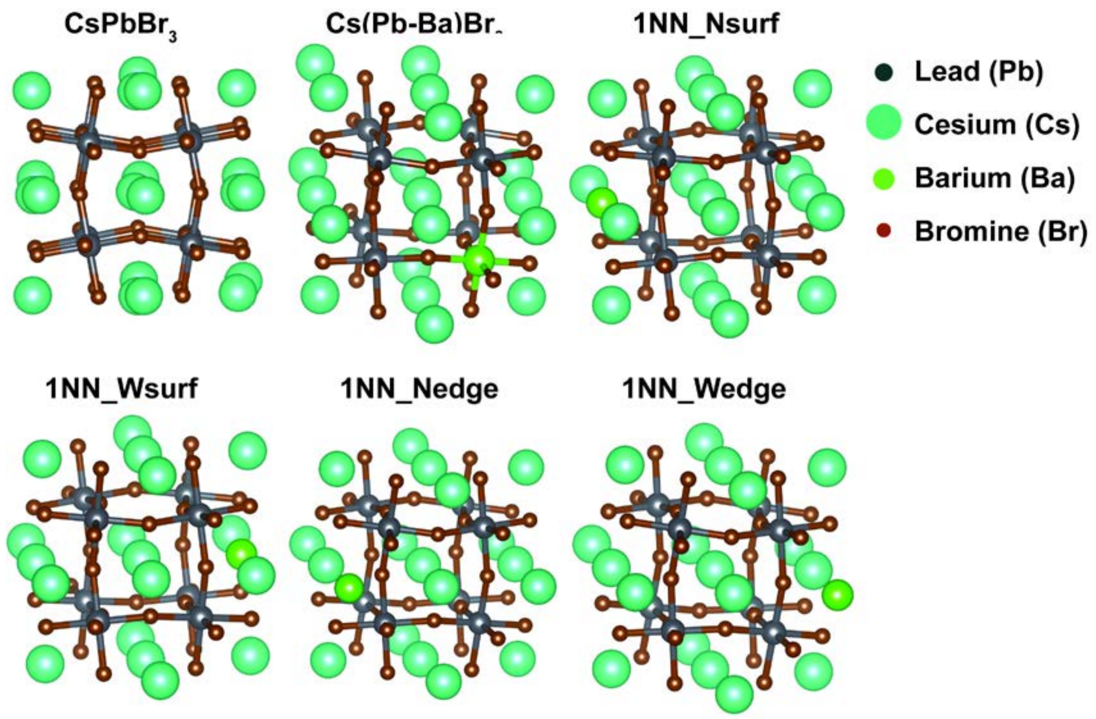

Supplementary Figure 19. Orthorhombic (*Pnma*) $CsBaPbBr_3$ clusters used for DFT ^{133}Cs chemical shift calculations.

Supplementary Table 1. DFT calculated ^{133}Cs chemical shieldings and referenced chemical shifts of the $CsBaPbBr_3$ clusters.

System	Description	Calculated ^{133}Cs chemical shielding [pm]	Calculated ^{133}Cs chemical shift [ppm]	Experimental ^{133}Cs chemical shift [ppm]
**Reference systems**				
(tetragonal) rt-$CsPbCl_3$	(tetragonal) rt-$CsPbCl_3$ cluster	6390.8	60.9	71
(hexagonal) δ-$CsPbI_3$	(hexagonal) δ-$CsPbI_3$ cluster	5940.4	235.5	240
(orthorhombic) rt-$CsPbBr_3$	(orthorhombic) rt-$CsPbBr_3$ cluster	6249.2	115.8	100.5
**Structures based on the cubic (*Pm-3m*) structure of $CsPbBr_3$**				
$Cs_{20}Pb_8Br_{36}$ ($CsPbBr_3$)	unperturbed cubic $CsPbBr_3$ cluster	6459.67	34.2	-
$Cs_{20}BaPb_7Br_{36}$ (Cs(Pb-Ba)Br_3)	Cs centered with one surrounding Pb replaced by Ba	6451.21	37.5	-
$BaCs_{18}Pb_8Br_{36}$ (1NN_edge)	1 nearest neighbor Cs replaced by Ba on adjacent edge	6455.86	35.7	-
$BaCs_{18}Pb_8Br_{36}$ (1NN_surf)	1 nearest neighbor Cs replaced by Ba on adjacent surface	6449.57	38.1	-
$Ba_2Cs_{16}Pb_8Br_{36}$ (2NN_surfO)	2 nearest neighbor Cs replaced by Ba on opposite surfaces	6438.06	42.6	-
$Ba_2Cs_{16}Pb_8Br_{36}$ (2NN_surfA)	2 nearest neighbor Cs replaced by Ba on adjacent surfaces	6436.44	43.2	-
$Ba_2Cs_{16}Pb_8Br_{36}$ (2NN_edge)	2 nearest neighbor Cs replaced by Ba on opposite edges	6452.22	37.1	-
$Ba_3Cs_{14}Pb_8Br_{36}$ (3NN_surfP)	3 nearest neighbor Cs replaced by Ba on	6428.11	46.5	-

	adjacent surfaces in a plane			
$Ba_3Cs_{14}Pb_8Br_{36}$ (3NN_surfC)	3 nearest neighbor Cs replaced by Ba on adjacent surfaces around a corner	6426.40	47.1	-
**Structures based on the orthorhombic (*Pnma*) room temperature structure of $CsPbBr_3$**				
$Cs_{20}Pb_8Br_{36}$ ($CsPbBr_3$)	unperturbed orthorhombic $CsPbBr_3$ cluster	6249.1	115.8	
$Cs_{20}BaPb_7Br_{36}$ ($Cs(Pb\text{-}Ba)Br_3$)	Cs centered with one surrounding Pb replaced by Ba	6241.8	118.7	
$BaCs_{18}Pb_8Br_{36}$ (1NN_Nsurf)	1 nearest neighbor Cs replaced by Ba on narrow adjacent surface	6245.7	117.2	
$BaCs_{18}Pb_8Br_{36}$ (1NN_Wsurf)	1 nearest neighbor Cs replaced by Ba on wide adjacent surface	6244.5	117.6	
$BaCs_{18}Pb_8Br_{36}$ (1NN_Nedge)	1 nearest neighbor Cs replaced by Ba on narrow adjacent edge	6247.0	116.6	
$BaCs_{18}Pb_8Br_{36}$ (1NN_Wedge)	1 nearest neighbor Cs replaced by Ba on wide adjacent edge	6244.5	117.6	

Supplementary Table 2. *J-V* parameters of $CsPbI_2Br$ based PSCs with different amount of BaI_2 as excess additive (Measurement was conducted under 100 mW cm^{-2} illumination with active area of 0.16 cm^{-2}).

Barium molar fraction, *x*	V_{OC} (V)	J_{SC} (mA cm^{-2})	FF (%)	PCE (%)
0	1.12	13.4	73.8	11.1
0.1	1.27	13.8	72.1	12.7
0.2	1.24	14.5	56.7	10.2

Supplementary Table 3. EDS analysis to identify the chemical composition of the bright dots and dark area from Fig. 4d.

Atom	Cs (%)	Pb (%)	I (%)	Br (%)	Ba (%)
Light-Calculation-Similar	18.9	10.9	38.4	22.6	9.2
Dark-Calculation	20.4	17.5	40.3	19.4	2.4

Supplementary Table 4. DFT calculated ^{137}Ba EFG tensor parameters of the $CsBaPbBr_3$ clusters.

**System**	**NQCC [MHz]**	**asymmetry parameter η**
$Cs_{54}BaPb_{26}Br_{108}$ (*Pb-Ba_large*)	2.26092	0.01994
$Cs_{22}BaPb_6Br_{36}$ (*Pb-Ba_small*)	16.2758	0.00649
$BaCs_{18}Pb_8Br_3$ (*Cs-Ba*)	0.516E-3	0.2584

Supplementary Methods

Computational details

[137]Ba EFG tensor calculations

We start from the assumption, that the ^{137}Ba cation is incorporated into the $CsPbBr_3$ lattice at either the A- or B-site replacing the Cs or Pb atom, respectively, without significantly changing the perovskite lattice formed by the $[PbBr_6]^{4-}$ octahedra.

We assemble the final clusters from $CsPbBr_3$ structure as $Cs_{54}BaPb_{26}Br_{108}$ (denoted as "*Pb-Ba_large*"), $Cs_{22}BaPb_6Br_{36}$ (denoted as "*Pb-Ba_small*") and $Cs_{18}BaPb_8Br_3$ (denoted as "*Cs-Ba*"), analogously to the ones used in the previous paper by *Kubicki et al.*,[1] ensuring charge compensation, high symmetry and direct comparability of the results. The clusters are shown in Supplementary Figure 17 and all of the structures are given in the Amsterdam Density Functional (ADF) [2,3] suite input and output format in the zip-file 137Ba_efg.zip. We use the highly symmetric cubic (*Pm-3m*) structure of $CsPbBr_3$ to simplify the cluster assembly. We expect the results to carry over to the orthorhombic (*Pnma*) room temperature structure observed experimentally.

For the EFG tensor calculations we used the ADF[2,4] suite within the density functional theory (DFT) framework. For the calculations we employed the GGA BP86[3,5] functional including the Grimme[6] dispersion correction and relativistic effects up to spin-orbit couplings within the ZORA[7-9] approximation. We used all-electron triple-ζ basis sets with two polarization functions (TZ2P).[10]

For all of the investigated systems the DFT calculated EFG tensor parameters are given in Supplementary Table 4.

Note, that the calculated EFG tensors from Pb-Ba_large and Pb-Ba_small should be the same, as they represent the same system. However, from Supplementary Table 4 it is evident, that the asymmetry parameter and especially the NQCC value differ significantly. This shows the importance of using large enough clusters so as to minimize the effect of symmetry breaking and the influence of atoms outside of the included coordination shells. Here, we choose Pb-Ba_large as estimator for the experimental data, as the larger cluster more accurately describes the central ^{137}Ba atom and its coordination. We also note, that for even larger clusters with an increased symmetry we expect an even smaller asymmetry parameter and NQCC. However, computationally, a larger cluster is not feasible or would require the use of less accurate basis-sets. Thus, the values calculated here should only be considered as an upper limit, rather than a quantitative measure.

[133]Cs chemical shift calculations

We start from the assumption, that the ^{137}Ba cation is incorporated into the $CsPbBr_3$ lattice at either the A- or B-site replacing the Cs or Pb atom respectively, without significantly changing the perovskite lattice formed by the $[PbBr_6]^{4-}$ octahedra.

As starting point we use both the highly symmetric cubic (*Pm-3m*) structure of $CsPbBr_3$ and the orthorhombic (*Pnma*) room temperature structure observed experimentally.

We assemble an unperturbed cluster from the $CsPbBr_3$ structure as $Cs_{20}Pb_8Br_{36}$ (denoted as "*$CsPbBr_3$*"), analog to the ones used in the previous paper by *Kubicki et al.*,[1] ensuring charge compensation, high symmetry and a direct comparability of the results. Subsequently, we

generate perturbed structures by replacing one to three of the non-central Cs atoms (or one of the Pb atoms) with Ba atoms, while maintaining the charge neutrality of the system.

All of the clusters are shown in Supplementary Figure 18 and 19 and all of the structures are given in the ADF [2,3] suite input and output format in the zip-file 133Cs_chemical_shift.zip. A short description of the different clusters in given in Supplementary Table 1**.**

For the chemical shielding calculations we used the ADF[2 4] suite within the DFT framework. For the calculations we employed the GGA BP86[3,5] functional including the Grimme[6] dispersion correction and relativistic effects up to spin-orbit couplings within the ZORA[7-9] approximation. We used all-electron triple-ζ basis sets with two polarization functions (TZ2P).[10]

The calculated ^{133}Cs magnetic shieldings (σ) were referenced to chemical shifts (δ) using the relation $\delta = a - b.\sigma$. The offset (a=2538) and slope (b=0.388) were calculated through a linear regression using the calculated and experimental chemical shifts of (hexagonal) δ-$CsPbI_3$, (tetragonal) rt-$CsPbCl_3$ and (orthorhombic) rt-$CsPbBr_3$.[11-13]

For all of the investigated systems the DFT ^{133}Cs calculated magnetic shieldings and shifts are given in Supplementary Table 1**.**

Structure optimization

We further investigate the symmetry preservation of the more chemically probable and energetically more stable structure featuring Ba incorporation, whereby Ba replaces Pb on the B-site. To that end, we generated a 2x2x2 periodic supercell with one Pb replaced by a Ba atom. Next, we optimized the positions of all the atoms using a periodic system within the DFT framework and the generalized gradient approximation (GGA) functional PBE[14] within the Quantum Espresso suite.[15] The DFT optimization includes the Grimme [6] dispersion correction and relativistic effects up to spin-orbit couplings. For every calculation we use a plane-wave maximum cutoff energy of 100 E_{Ryd} and a 2x2x2 Monkhorst-Pack[16] grid of k-points. The energy convergence threshold was set to 10^{-4} Ry and the force convergence threshold was set to 10^{-3} F_{Ryd}.

The optimization lead to no significant changes within the structure, with an all atom root-mean-square deviation below 0.03 Å. All the QE output and input files are given in the zip-file relaxation.zip.

Supplementary References

1 Kubicki, D. J. *et al.* Cation dynamics in mixed-cation $(MA)_x(FA)_{1-x}PbI_3$ hybrid perovskites from solid-state NMR. *J. Am. Chem. Soc.* **139**, 10055-10061 (2017).

2 Guerra, C. F., Snijders, J. G., te Velde, G. & Baerends, E. J. Towards an order-N DFT method. *Theor. Chem. Acc.* **99**, 391-403 (1998).

3 Perdew, J. P. Density-functional approximation for correlation-energy of the inhomogeneous electron-gas. *Phys. Rev. B* **33**, 8822-8824 (1986).

4 te Velde, G. *et al.* Chemistry with ADF. *J. Comput. Chem.* **22**, 931-967 (2001).

5 Becke, A. D. Density-functional exchange-energy approximation with correct asymptotic-behavior. *Phys. Rev. A* **38**, 3098-3100 (1988).

6 Grimme, S. Semiempirical GGA-type density functional constructed with a long-range dispersion correction. *J. Comput. Chem.* **27**, 1787-1799 (2006).

7 Vanlenthe, E., Baerends, E. J. & Snijders, J. G. Relativistic tota-energy using regular approximations. *J. Chem. Phys.* **101**, 9783-9792 (1994).

8 Vanlenthe, E., Baerends, E. J. & Snijders, J. G. Relativistic regular 2-component hamiltonians. *J. Chem. Phys.* **99**, 4597-4610 (1993).

9 van Lenthe, E., Ehlers, A. & Baerends, E. J. Geometry optimizations in the zero order regular approximation for relativistic effects. *J. Chem. Phys.* **110**, 8943-8953 (1999).

10 Van Lenthe, E. & Baerends, E. J. Optimized slater-type basis sets for the elements 1-118. *J. Comput. Chem.* **24**, 1142-1156 (2003).

11 Kubicki, D. J. *et al.* Phase segregation in Cs-, Rb- and K-doped mixed-cation $(MA)_x(FA)_{1-x}PbI_3$ hybrid perovskites from solid-state NMR. *J. Am. Chem. Soc.* **139**, 14173-14180 (2017).

12 Moller C.K. The structure of perovskite-like caesium plumbo trihalides. *Mat. Fys. Medd. K. Dan. Vidensk. Selsk.* **32**, 1-27 (1959)

13 Rodova, M., Brozek, J., Knizek, K. & Nitsch, K. Phase transitions in ternary caesium lead bromide. *J. Therm. Anal. Calorim.* **71**, 667-673 (2003).

14 Perdew, J. P., Burke, K. & Ernzerhof, M. Generalized gradient approximation made simple (vol 77, pg 3865, 1996). *Phys. Rev. Lett.* **78**, 1396-1396 (1997).

15 Giannozzi, P. *et al.* Quantum espresso: a modular and open-source software project for quantum simulations of materials. *J. Phys.-Condes. Matter* **21**, 395502 (2009).

16 Pack, J. D. & Monkhorst, H. J. Special points for brillouin-zone integrations-reply. *Phys. Rev. B* **16**, 1748-1749 (1977).
