# Supplementary material for: Ba-induced phase segregation and band gap reduction in mixed-halide inorganic perovskite solar cells
Source: Nat Commun. 2019 Oct 15;10:4686. doi: 10.1038/s41467-019-12678-5 (PMC6794321; doi:10.1038/s41467-019-12678-5)
Supplement: Supplementary file 2 — Solar Cells Reporting Summary [file 41467_2019_12678_MOESM2_ESM.pdf]

nature research

Corresponding author(s): Anders Hagfeldt, Wanchun Xiang

☐ Initial submission ☐ Revised version ☒ Final submission

# Solar Cells Reporting Summary

Nature Research wishes to improve the reproducibility of the work that we publish. This form is intended for publication with all accepted papers reporting the characterization of photovoltaic devices and provides structure for consistency and transparency in reporting. Some list items might not apply to an individual manuscript, but all fields must be completed for clarity.

For further information on Nature Research policies, including our data availability policy, see Authors & Referees.

## Experimental design

**Please check: are the following details reported in the manuscript?**

### 1. Dimensions

Area of the tested solar cells

☒ Yes ☐ No

The active area of solar cells is 0.16 cm2. "Photovoltaic performance. " in “Methods” section

Explain why this information is not reported/not relevant.

Method used to determine the device area

☒ Yes ☐ No

A black metal mask with an area of 0.16 cm-2 was applied to define the active area of the cells and to avoid the overestimation of the light input. "Photovoltaic performance. " in “Methods” section

Explain why this information is not reported/not relevant.

### 2. Current-voltage characterization

Current density-voltage (J-V) plots in both forward and backward direction

☐ Yes ☒ No

State where this information can be found in the text.

Not relevant

Voltage scan conditions
*For instance: scan direction, speed, dwell times*

☒ Yes ☐ No

JV curves were measured with a scanning rate of 10 mV/s (voltage step of 5 mV and no delay time). "Photovoltaic performance. " in “Methods” section

Not relevant

Test environment
*For instance: characterization temperature, in air or in glove box*

☒ Yes ☐ No

JV measurements were carried out in air at room temperature . "Photovoltaic performance. " in “Methods” section

Not relevant

Protocol for preconditioning of the device before its characterization

☐ Yes ☒ No

State where this information can be found in the text.

Not relevant

Stability of the J-V characteristic
*Verified with time evolution of the maximum power point or with the photocurrent at maximum power point; see ref. 7 for details.*

☒ Yes ☐ No

Fig. 2b and FigS13. In "Device stability " section and also in the "Stability" of "Methods" section

Explain why this information is not reported/not relevant.

### 3. Hysteresis or any other unusual behaviour

Description of the unusual behaviour observed during the characterization

☒ Yes ☐ No

The whole manuscript is reporting a finding of phase segregation upon Ba addition

Explain why this information is not reported/not relevant.

Related experimental data

☒ Yes ☐ No

The whole manuscript

Explain why this information is not reported/not relevant.

### 4. Efficiency

External quantum efficiency (EQE) or incident photons to current efficiency (IPCE)

☒ Yes ☐ No

FIg. 2c

Explain why this information is not reported/not relevant.

A comparison between the integrated response under the standard reference spectrum and the response measure under the simulator

☒ Yes ☐ No

The integrated Jsc of 14.1 mA cm-2 agrees also with the measured value. Fig.2b

Explain why this information is not reported/not relevant.

| | | |
|---|---|---|
| For tandem solar cells, the bias illumination and bias voltage used for each subcell | ☐ Yes<br>☒ No | State where this information can be found in the text.<br>Our devices were only fabricated for single solar cells. |

## 5. Calibration

| | | |
|---|---|---|
| Light source and reference cell or sensor used for the characterization | ☒ Yes<br>☐ No | "Photovoltaic performance. " in "Methods" section<br>Explain why this information is not reported/not relevant. |
| Confirmation that the reference cell was calibrated and certified | ☒ Yes<br>☐ No | The reference Si photodiode was calibrated by Schott. "Photovoltaic performance. " in "Methods" section<br>Explain why this information is not reported/not relevant. |
| Calculation of spectral mismatch between the reference cell and the devices under test | ☐ Yes<br>☒ No | Mismatch factor of 1 was used in our measurements<br>Not relevant |

## 6. Mask/aperture

| | | |
|---|---|---|
| Size of the mask/aperture used during testing | ☒ Yes<br>☐ No | 0.16 cm2 "Photovoltaic performance. " in "Methods" section<br>Explain why this information is not reported/not relevant. |
| Variation of the measured short-circuit current density with the mask/aperture area | ☐ Yes<br>☒ No | State where this information can be found in the text.<br>Not relevant |

## 7. Performance certification

| | | |
|---|---|---|
| Identity of the independent certification laboratory that confirmed the photovoltaic performance | ☐ Yes<br>☒ No | State where this information can be found in the text.<br>We did not certify our cells. |
| A copy of any certificate(s)<br>*Provide in Supplementary Information* | ☐ Yes<br>☒ No | State where this information can be found in the text.<br>We did not certify our cells. |

## 8. Statistics

| | | |
|---|---|---|
| Number of solar cells tested | ☐ Yes<br>☒ No | State where this information can be found in the text.<br>Not relevant |
| Statistical analysis of the device performance | ☐ Yes<br>☒ No | State where this information can be found in the text.<br>Not relevant |

## 9. Long-term stability analysis

| | | |
|---|---|---|
| Type of analysis, bias conditions and environmental conditions<br>*For instance: illumination type, temperature, atmosphere humidity, encapsulation method, preconditioning temperature* | ☒ Yes<br>☐ No | "Stability" of "Methods" section. Fig. S13<br>Explain why this information is not reported/not relevant. |

## Further reading


1. Shrotriya, V. *et al*. Accurate measurement and characterization of organic solar cells. *Adv. Funct. Mater.* **16**, 2016–2023 (2006).
2. Dennler, G. *et al*. The value of values. *Mat. Today* **10**, 56 (2007).
3. Cravino, A., Schilinsky, P. & Brabec, C. J. Characterization of organic solar cells: the importance of device layout. *Adv. Funct. Mater.* **17**, 3906–3910 (2007).
4. Reese, M. O. *et al*. Consensus stability testing protocols for organic photovoltaic materials and devices. *Sol. Energ. Mat. Sol. C* **95**, 1253–1267 (2011).
5. Snaith H. .J. The perils of solar cell efficiency measurements. *Nat. Photon*. **6**, 337–340 (2012).
6. Luber, E. J. & Buriak, J. M. Reporting performance in organic photovoltaic devices. *ACS Nano* **7**, 4708–4714 (2013).
7. Snaith, H. J. *et al*. Anomalous hysteresis in perovskite solar cells. *J. Phys. Chem. Lett.* **5**, 1511–1515 (2014).
8. Grätzel M. The light and shade of perovskite solar cells. *Nat. Mat*. **13**, 838–842 (2014).
9. Zimmermann E. *et al*. Erroneous efficiency reports harm organic solar cell research. *Nat. Photon*. **8**, 669–672 (2014).
10. Beard M.C., Luther J.M. & Nozik A.J. The promise and challenge of nanostructured solar cells. *Nat. Nanotech*. **9**, 951–954 (2014).
11. Timmreck, R. *et al*. Characterization of tandem organic solar cells. *Nat. Photon.* **9**, 478–479 (2015).


A number of international committees develop industry standards on the characterization of photovoltaic technologies (for example ASTM-E44 and IEC-TC 82), which can provide guidance for academic research.

This checklist template is licensed under a Creative Commons Attribution 4.0 International License, which permits use, sharing, adaptation, distribution and reproduction in any medium or format, as long as you give appropriate credit to the original author(s) and the source, provide a link to the Creative Commons license, and indicate if changes were made. The images or other third party material in this article are included in the article's Creative Commons license, unless indicated otherwise in a credit line to the material. If material is not included in the article's Creative Commons license and your intended use is not permitted by statutory regulation or exceeds the permitted use, you will need to obtain permission directly from the copyright holder. To view a copy of this license, visit http://creativecommons.org/licenses/by/4.0/
